# Military and Medical Recruiting

**Published:** 1915-11-06

**Authors:** 


### The Hospital

The Workers' Newspaper of Administrative Medicine and Institutional Life, Administration, National Insurance and Health.

No. 1534, Vol. LIX. SATURDAY, NOVEMBER 6, 1915. PRICE ONE PENNY.

### MILITARY AND MEDICAL RECRUITING.

The controversy between the advocates of conscription on the one hand and of voluntary enlistment on the other has presented many unedifying features, though possibly these have not been more numerous or more gross than is frequent in popular controversies in general. But the great issues which are at stake in the present war give to all the questions connected with it a special prominence and sharpen and emphasise both opinion and the manner of expressing it. Whatever evils have attended the dispute this has apparently had the advantage of forcing the question to the point of decision, and in the circumstances of the hour decision is a quality of high merit. Right and wise decision, if possible, but almost any clear choice is to be preferred to continued uncertainty and agitation. The commanding fact from this point of view, as may be gathered from recent announcements, is that the country is under a solemn pledge to its Allies to raise a given number of soldiers for the purposes of the war. The good faith of the nation is, therefore, involved, and it cannot be doubted that in one way or another the pledge will be redeemed. How far the more recent efforts to this end are proving successful can only be surmised from statements which have appeared in the public Press, and our readers must judge these for themselves. But assuming our conclusion to be justified, and we believe it to be beyond dispute, the decision to enrol men at the rate of thirty thousand a week implies a claim which the medical profession cannot possibly ignore.

As the national word has been given to find still more soldiers, and as soldiers cannot be sent into the field without a proper and adequate medical equipment, it follows inevitably that, as a State necessity, doctors must be found for the new Armies. And we are afraid we must add that, if voluntary enlistment does not secure them, other methods will be put into operation. What is at stake is nothing less than the safety of the country, and in view of this the preferences, the convenience, and even the welfare of individuals become of secondary moment. Whatever the personal sacrifice it now takes the stern form of duty. It must be freely admitted that the sacrifice demanded from the junior members of the profession engaged in private practice is a very considerable one. Still younger men who have but recently obtained their diplomas have no grounds for hesitation in responding to the country's call. But a man who has already settled in practice and who has also definite domestic responsibilities may reasonably feel that the demand made upon him is an extremely heavy one. In spite of the desire on the part of his colleagues and others to protect his interests, service with the Army must inevitably mean for him loss of income and, what is even a more trying experience, loss of opportunity for the extension of his usefulness and for the gratification of his legitimate ambitions. Nothing but the supreme necessity, the safety of the State, can justify such a summons. This necessity, however, exists, and it is the motive which commands the whole of the present situation. It is quite true that many, to their honour be it spoken, have already recognised the seriousness of the situation and have risked everything—and not a few have sacrificed everything—for the national cause. To do much, however, is not sufficient; it is necessary to do enough, and the term has to be construed in harmony with hard facts. One of these facts is that the Army needs and will for some time continue to need more doctors, for without these the duty of the nation cannot be acquitted. This is the consideration which it is necessary to keep prominently before the profession, and whatever be the sacrifice involved it is essential that the obligation be fully discharged.

With the aims and methods of the Central Medical War Committee we have previously expressed our sympathy, and we know that, at least in some districts, the work of the committee has met with considerable success. Our one desire is to help the committee to retain the recruiting of medical men in its hands. So far as the Army authorities at headquarters are concerned, this is the wish of those in command. But there are not wanting evidences that local recruiting committees, acting on the information obtained under the Registration Act, are disposed, at least in certain districts, to include medical practitioners in the scheme of their operations. Manifestly such a proceeding is much to be deprecated, for these committees are quite unsuited to deal with the delicate questions which must arise when a medical man has to leave his practice and other interests to the care of his colleagues. It is in the interest both of the individual and of the profession that all such matters should be regulated by competent and sympathetic persons who are fitted by personal experience to deal with them. Advice and help on all points are now readily to be obtained for the asking, and there is no advantage, quite the contrary indeed, in permitting or encouraging interference from the outside. But if this domestic arrangement is to be maintained it must be able to show a quite unchallenged success. Behind everything lies the national need, and while professional machinery is desirable it is rather the end than the means of which the authorities are thinking. No one can wish to see the recruiting of the medical profession by lay committees, and still less to see doctors enlisted under compulsion. Our confidence is that neither of these methods will be necessary, but if they are to be avoided willing service must be offered in still increasing measure.

